# Crystal structure of bis­{μ-2-[(di­methyl­amino)­meth­yl]ferrocene­seleno­lato}bis[chlorido­palladium(II)]

**DOI:** 10.1107/S1600536814019503

**Published:** 2014-09-03

**Authors:** Esther M. Takaluoma, Raija Oilunkaniemi, Risto S. Laitinen

**Affiliations:** a Department of Chemistry, P.O. Box 3000, FI-90014 University of Oulu, Finland

**Keywords:** crystal structure, dinuclear palladium complex, ferrocene­seleno­lato ligand

## Abstract

The dinuclear title compound, [PdCl{Se[(C_5_H_5_)Fe(C_5_H_3_)_2_CH_2_N(CH_3_)_2_]}]_2_ was obtained by the reaction of [PdCl_2_(NCPh)_2_] with 2-[(*N*,*N*′-di­methyl­amino)­meth­yl]ferro­cene­seleno­late and the crystals for the structure determination were grown from a mixture of THF and *n*-hexane. Both Pd^II^ atoms are coordinated by the bridging Se atoms and by the amino N atoms of the bidentate 2-[(*N*,*N*′-di­methyl­amino)­meth­yl]ferrocene­seleno­late ligand, as well as by Cl atoms, and show a distorted square-planar coordination. The angle between the Pd—Se—Se planes of the two Pd atoms is 149.31 (3)°. Weak Cl⋯H hydrogen bonds link the binuclear complexes into a three-dimensional network.

## Related literature   

The structural data for mononuclear [PdCl(C_9_H_12_NSe)PPh_3_] containing a chelating 2-[(*N*,*N*′-di­methyl­amino)­meth­yl]benzene­seleno­late ligand have been reported by Takaluoma *et al.* (2014 ▶). For the synthesis of a related dinuclear palladium complex containing a chiral 2-[(*N*,*N*′-di­methyl­amino)­eth­yl]ferrocene­seleno­late ligand, see: Kaur *et al.* (2009 ▶). For the structure of the dinuclear palladium complex [PdCl(C_9_H_12_NSe)]_2_, see: Chakravorty *et al.* (2012 ▶); Pop *et al.* (2013 ▶). For the synthesis of lithium [2-(*N*,*N*′-di­methyl­amino)­meth­yl]ferrocene­seleno­late, see: Gornitzka *et al.* (1992 ▶).
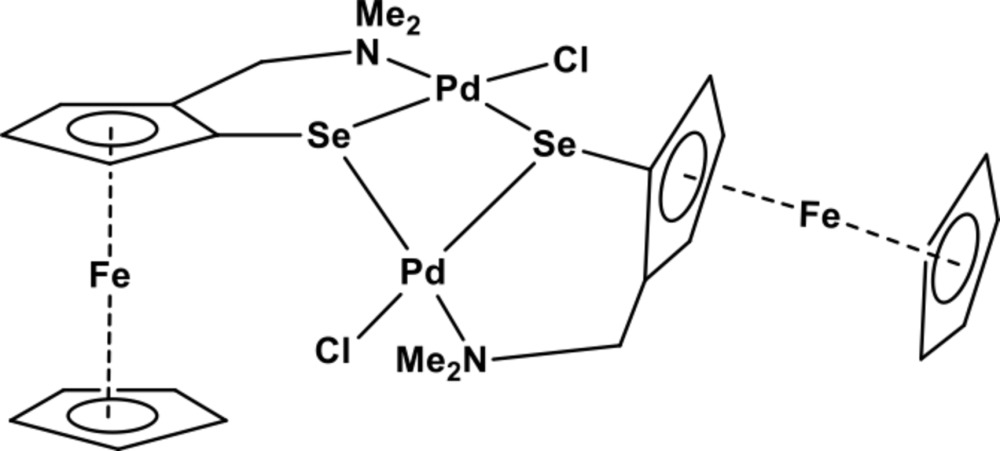


## Experimental   

### Crystal data  

[Fe_2_Pd_2_(C_5_H_5_)_2_Cl_2_(C_8_H_11_NSe)_2_]
*M* *_r_* = 925.85
Monoclinic, 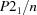

*a* = 13.030 (3) Å
*b* = 10.985 (2) Å
*c* = 19.925 (4) Å
β = 93.25 (3)°
*V* = 2847.4 (10) Å^3^
*Z* = 4
Mo *K*α radiation
μ = 5.01 mm^−1^
*T* = 120 K
0.40 × 0.05 × 0.05 mm

### Data collection  

Bruker–Nonius KappaCCD diffractometer
Absorption correction: multi-scan (*XPREP* in *SHELXTL*; Sheldrick, 2008 ▶) *T*_min_ = 0.655, *T*_max_ = 0.938
19836 measured reflections
4847 independent reflections
3937 reflections with *I* > 2σ(*I*)
*R*_int_ = 0.078

### Refinement  

*R*[*F* ^2^ > 2σ(*F* ^2^)] = 0.039
*wR*(*F* ^2^) = 0.088
*S* = 1.07
4847 reflections
330 parameters
H-atom parameters constrained
Δρ_max_ = 0.69 e Å^−3^
Δρ_min_ = −0.62 e Å^−3^

### 

Data collection: *COLLECT* (Bruker, 2008 ▶); cell refinement: *DENZO-SMN* (Otwinowski & Minor, 1997 ▶); data reduction: *DENZO-SMN*; program(s) used to solve structure: *SIR92* (Altomare *et al.*, 1993 ▶); program(s) used to refine structure: *SHELXL2013* (Sheldrick, 2008) ▶; molecular graphics: *DIAMOND* (Brandenburg, 2006 ▶); software used to prepare material for publication: *WinGX* (Farrugia, 2012 ▶).

## Supplementary Material

Crystal structure: contains datablock(s) I, global. DOI: 10.1107/S1600536814019503/zl2600sup1.cif

Structure factors: contains datablock(s) I. DOI: 10.1107/S1600536814019503/zl2600Isup2.hkl

**Fig. 1.**
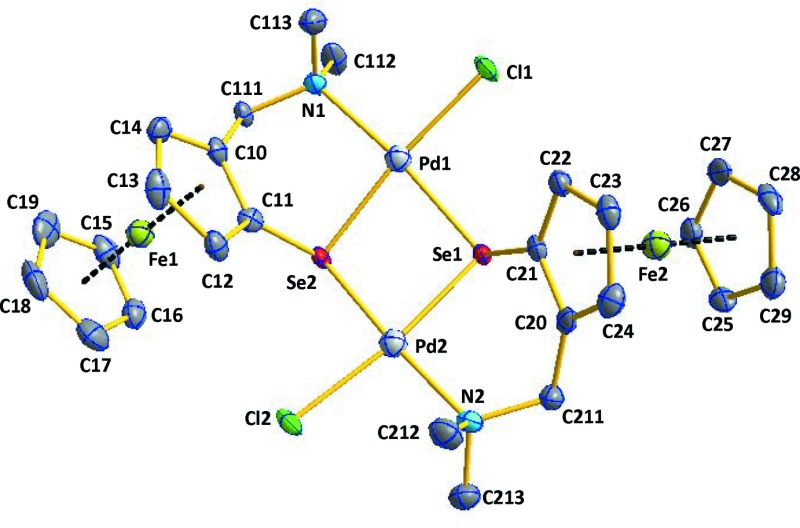
The molecular structure of the title compound indicating the numbering of the atoms. The thermal ellipsoids have been drawn at 50% probability. Hydrogen atoms have been omitted for clarity.

. DOI: 10.1107/S1600536814019503/zl2600fig1.tif
The mol­ecular structure of the title compound indicating the numbering of the atoms. The thermal ellipsoids have been drawn at 50% probability. Hydrogen atoms have been omitted for clarity.

CCDC reference: 1021634

Additional supporting information:  crystallographic information; 3D view; checkCIF report

## Figures and Tables

**Table:** 

Pd1—N1	2.182 (5)
Pd1—Cl1	2.3585 (17)
Pd1—Se2	2.3898 (8)
Pd1—Se1	2.4051 (9)
Pd2—N2	2.152 (5)
Pd2—Cl2	2.3540 (17)
Pd2—Se2	2.3716 (8)
Pd2—Se1	2.4166 (8)

**Table:** 

N1—Pd1—Cl1	92.99 (15)
N1—Pd1—Se2	93.62 (14)
Cl1—Pd1—Se2	173.39 (5)
N1—Pd1—Se1	172.87 (15)
Cl1—Pd1—Se1	93.59 (5)
Se2—Pd1—Se1	79.80 (3)
N2—Pd2—Cl2	92.05 (15)
N2—Pd2—Se2	175.53 (15)
Cl2—Pd2—Se2	91.22 (5)
N2—Pd2—Se1	96.84 (14)
Cl2—Pd2—Se1	171.10 (5)
Se2—Pd2—Se1	79.92 (3)

**Table 2:** Hydrogen-bond geometry (Å, °)

*D*—H⋯*A*	*D*—H	H⋯*A*	*D*⋯*A*	*D*—H⋯*A*
C113—H11*F*⋯Cl1	0.98	2.70	3.347 (8)	124
C212—H21*D*⋯Cl2	0.98	2.75	3.394 (8)	124
C213—H21*H*⋯Cl2	0.98	2.74	3.384 (8)	124
C22—H22⋯Cl1	0.95	2.82	3.537 (7)	133

## supplementary crystallographic information

### S1. Synthesis and crystallization

Lithium [2-(*N,'N*-di­methyl­amino)­methyl]­ferrocene­seleno­late was obtained, as described by Gornitzka *et al.* (1992). Li­thia­ted *N,'N*-di­methyl­amino­methyl­ferrocene (0.100 g, 0.40 mmol) was dissolved in 30 ml of THF and cooled to -78 °C. Selenium (0.030 g 0.38 mmol) was added into the solution and the reaction solution was warmed to room temperature. The light yellow solution was added to 0.144 g (0.38 mmol) of [PdCl_2_(NCPh)_2_] in 30 ml THF. The solution turned dark red and was stirred over night and was subsequently evaporated to 5 ml. A dark red powder was obtained by precipitation with *n*-hexane. It was washed twice with *n*-hexane and dried. Both mass spectrometry and elemental analysis indicated that the powder was a mixture. A small amount of dark red crystals of the title compound, which were suitable for crystal structure determination, were grown from a mixture of THF and *n*-hexane.

### S2. Refinement

Crystal data, data collection and structure refinement details are summarized below. H atoms were positioned geometrically and refined using a riding model. The C—H fixed bond lengths are 0.98, 0.99, and 0.95 Å for methyl, methyl­ene, and aromatic hydrogens, respectively. *U*_iso_(H) was constrained to be 1.5 times *U*_eq_(C) for methyl hydrogens and 1.2 times *U*_eq_(C) for methyl­ene and aromatic hydrogens.

### Crystal data

**Table:**

[Fe_2_Pd_2_(C_5_H_5_)_2_Cl_2_(C_8_H_11_NSe)_2_]	*F*(000) = 1792
*M**_r_* = 925.85	*D*_x_ = 2.160 Mg m^−^^3^
Monoclinic, *P*2_1_/*n*	Mo *K*α radiation, λ = 0.71073 Å
*a* = 13.030 (3) Å	Cell parameters from 3937 reflections
*b* = 10.985 (2) Å	θ = 3.1–25.0°
*c* = 19.925 (4) Å	µ = 5.01 mm^−^^1^
β = 93.25 (3)°	*T* = 120 K
*V* = 2847.4 (10) Å^3^	Needle, red
*Z* = 4	0.40 × 0.05 × 0.05 mm

### Data collection

**Table:**

Bruker–Nonius KappaCCD diffractometer	3937 reflections with *I* > 2σ(*I*)
Radiation source: fine-focus sealed tube	*R*_int_ = 0.078
φ scans, and ω scans with κ offsets	θ_max_ = 25.0°, θ_min_ = 3.1°
Absorption correction: multi-scan (*XPREP* in *SHELXTL*; Sheldrick, 2008)	*h* = −15→15
*T*_min_ = 0.655, *T*_max_ = 0.938	*k* = −13→13
19836 measured reflections	*l* = −23→21
4847 independent reflections	

### Refinement

**Table:**

Refinement on *F*^2^	Secondary atom site location: difference Fourier map
Least-squares matrix: full	Hydrogen site location: inferred from neighbouring sites
*R*[*F*^2^ > 2σ(*F*^2^)] = 0.039	H-atom parameters constrained
*wR*(*F*^2^) = 0.088	*w* = 1/[σ^2^(*F*_o_^2^) + (0.0248*P*)^2^ + 17.5058*P*] where *P* = (*F*_o_^2^ + 2*F*_c_^2^)/3
*S* = 1.07	(Δ/σ)_max_ = 0.001
4847 reflections	Δρ_max_ = 0.69 e Å^−^^3^
330 parameters	Δρ_min_ = −0.62 e Å^−^^3^
0 restraints	Extinction correction: *SHELXL2013* (Sheldrick, 2013), Fc^*^=kFc[1+0.001xFc^2^λ^3^/sin(2θ)]^-1/4^
Primary atom site location: structure-invariant direct methods	Extinction coefficient: 0.00086 (12)

### Special details

**Table:**

Geometry. All e.s.d.'s (except the e.s.d. in the dihedral angle between two l.s. planes) are estimated using the full covariance matrix. The cell e.s.d.'s are taken into account individually in the estimation of e.s.d.'s in distances, angles and torsion angles; correlations between e.s.d.'s in cell parameters are only used when they are defined by crystal symmetry. An approximate (isotropic) treatment of cell e.s.d.'s is used for estimating e.s.d.'s involving l.s. planes.

### Fractional atomic coordinates and isotropic or equivalent isotropic displacement parameters (Å^2^)

**Table:**

	*x*	*y*	*z*	*U*_iso_*/*U*_eq_	
Pd1	0.22704 (4)	0.80997 (4)	0.50395 (2)	0.01626 (14)	
Pd2	0.15556 (4)	1.11994 (4)	0.48597 (2)	0.01682 (14)	
Se1	0.10993 (5)	0.94796 (5)	0.55379 (3)	0.01595 (16)	
Se2	0.20573 (5)	0.95790 (5)	0.41633 (3)	0.01657 (16)	
Cl1	0.23531 (16)	0.67804 (16)	0.59748 (8)	0.0326 (4)	
Cl2	0.20566 (14)	1.26486 (15)	0.40718 (9)	0.0304 (4)	
N1	0.3262 (4)	0.6940 (5)	0.4467 (3)	0.0218 (12)	
N2	0.1156 (4)	1.2594 (5)	0.5557 (3)	0.0214 (12)	
Fe1	0.41999 (7)	1.00911 (9)	0.32407 (5)	0.0206 (2)	
Fe2	0.11295 (7)	1.00558 (8)	0.72807 (4)	0.0182 (2)	
C10	0.4069 (5)	0.8686 (6)	0.3912 (3)	0.0210 (14)	
C11	0.3504 (5)	0.9741 (6)	0.4102 (3)	0.0165 (13)	
C12	0.4197 (5)	1.0724 (6)	0.4203 (3)	0.0238 (15)	
H12	0.4028	1.1527	0.4334	0.029*	
C13	0.5182 (5)	1.0295 (7)	0.4073 (3)	0.0275 (16)	
H13	0.5792	1.0770	0.4103	0.033*	
C14	0.5132 (5)	0.9046 (6)	0.3891 (3)	0.0229 (15)	
H14	0.5690	0.8544	0.3777	0.028*	
C15	0.3522 (6)	0.9628 (7)	0.2323 (4)	0.0349 (18)	
H15	0.3164	0.8894	0.2216	0.042*	
C16	0.3081 (6)	1.0701 (7)	0.2564 (4)	0.0304 (17)	
H16	0.2376	1.0813	0.2644	0.037*	
C17	0.3859 (6)	1.1578 (7)	0.2667 (4)	0.0354 (18)	
H17	0.3775	1.2382	0.2830	0.042*	
C18	0.4799 (6)	1.1041 (8)	0.2481 (4)	0.041 (2)	
H18	0.5452	1.1427	0.2498	0.050*	
C19	0.4585 (6)	0.9825 (8)	0.2265 (4)	0.039 (2)	
H19	0.5067	0.9255	0.2111	0.047*	
C20	0.1446 (5)	1.1310 (6)	0.6565 (3)	0.0186 (14)	
C21	0.1723 (5)	1.0104 (6)	0.6354 (3)	0.0175 (13)	
C22	0.2458 (5)	0.9604 (6)	0.6829 (3)	0.0199 (14)	
H22	0.2761	0.8819	0.6810	0.024*	
C23	0.2657 (5)	1.0499 (6)	0.7341 (3)	0.0228 (15)	
H23	0.3116	1.0412	0.7726	0.027*	
C24	0.2051 (5)	1.1540 (6)	0.7174 (3)	0.0256 (16)	
H24	0.2046	1.2273	0.7427	0.031*	
C25	−0.0403 (5)	1.0176 (6)	0.7444 (3)	0.0252 (16)	
H25	−0.0886	1.0705	0.7219	0.030*	
C26	−0.0156 (5)	0.8971 (6)	0.7233 (3)	0.0269 (16)	
H26	−0.0435	0.8559	0.6846	0.032*	
C27	0.0596 (5)	0.8507 (6)	0.7720 (4)	0.0271 (16)	
H27	0.0900	0.7722	0.7714	0.033*	
C28	0.0807 (5)	0.9414 (7)	0.8208 (3)	0.0261 (16)	
H28	0.1283	0.9344	0.8585	0.031*	
C29	0.0190 (5)	1.0452 (7)	0.8045 (3)	0.0287 (16)	
H29	0.0177	1.1192	0.8292	0.034*	
C111	0.3567 (5)	0.7471 (6)	0.3814 (3)	0.0190 (14)	
H11A	0.4048	0.6909	0.3604	0.023*	
H11B	0.2949	0.7557	0.3505	0.023*	
C112	0.2682 (7)	0.5809 (6)	0.4316 (4)	0.0354 (19)	
H11C	0.3131	0.5217	0.4110	0.053*	
H11D	0.2092	0.5989	0.4005	0.053*	
H11E	0.2438	0.5471	0.4733	0.053*	
C113	0.4211 (6)	0.6654 (7)	0.4884 (4)	0.0310 (17)	
H11F	0.4029	0.6235	0.5295	0.047*	
H11G	0.4575	0.7411	0.5003	0.047*	
H11H	0.4655	0.6128	0.4629	0.047*	
C211	0.0708 (5)	1.2127 (6)	0.6178 (3)	0.0222 (14)	
H21A	0.0069	1.1671	0.6054	0.027*	
H21B	0.0527	1.2822	0.6465	0.027*	
C212	0.2097 (6)	1.3284 (6)	0.5740 (4)	0.0293 (16)	
H21C	0.1940	1.3935	0.6054	0.044*	
H21D	0.2365	1.3640	0.5334	0.044*	
H21E	0.2613	1.2738	0.5953	0.044*	
C213	0.0381 (6)	1.3448 (6)	0.5232 (4)	0.0305 (17)	
H21F	0.0235	1.4103	0.5547	0.046*	
H21G	−0.0255	1.3002	0.5110	0.046*	
H21H	0.0655	1.3796	0.4826	0.046*	

### Atomic displacement parameters (Å^2^)

**Table:**

	*U*^11^	*U*^22^	*U*^33^	*U*^12^	*U*^13^	*U*^23^
Pd1	0.0243 (3)	0.0136 (2)	0.0112 (2)	−0.0008 (2)	0.0030 (2)	0.00145 (18)
Pd2	0.0220 (3)	0.0142 (2)	0.0143 (3)	−0.0003 (2)	0.0020 (2)	0.00292 (19)
Se1	0.0195 (3)	0.0157 (3)	0.0128 (3)	−0.0016 (3)	0.0025 (3)	0.0013 (2)
Se2	0.0211 (3)	0.0171 (3)	0.0116 (3)	−0.0020 (3)	0.0013 (3)	0.0021 (2)
Cl1	0.0598 (12)	0.0218 (8)	0.0169 (8)	0.0077 (8)	0.0083 (8)	0.0085 (7)
Cl2	0.0450 (11)	0.0212 (8)	0.0257 (9)	−0.0012 (8)	0.0085 (8)	0.0115 (7)
N1	0.031 (3)	0.016 (3)	0.019 (3)	0.002 (2)	0.006 (2)	0.000 (2)
N2	0.026 (3)	0.015 (3)	0.023 (3)	0.001 (2)	0.001 (2)	0.004 (2)
Fe1	0.0224 (5)	0.0249 (5)	0.0147 (5)	−0.0055 (4)	0.0033 (4)	0.0018 (4)
Fe2	0.0183 (5)	0.0224 (5)	0.0141 (5)	0.0009 (4)	0.0029 (4)	0.0001 (4)
C10	0.025 (4)	0.026 (4)	0.012 (3)	−0.003 (3)	0.002 (3)	0.002 (3)
C11	0.017 (3)	0.020 (3)	0.012 (3)	−0.002 (3)	−0.006 (3)	−0.002 (3)
C12	0.030 (4)	0.026 (4)	0.016 (3)	−0.004 (3)	0.005 (3)	0.001 (3)
C13	0.028 (4)	0.039 (4)	0.015 (3)	−0.012 (3)	0.002 (3)	−0.006 (3)
C14	0.023 (4)	0.028 (4)	0.019 (3)	0.005 (3)	0.005 (3)	−0.001 (3)
C15	0.045 (5)	0.040 (5)	0.019 (4)	−0.011 (4)	−0.003 (3)	0.005 (3)
C16	0.028 (4)	0.041 (4)	0.023 (4)	0.000 (3)	0.004 (3)	0.006 (3)
C17	0.042 (5)	0.034 (4)	0.030 (4)	−0.004 (4)	−0.003 (4)	0.011 (3)
C18	0.032 (4)	0.057 (6)	0.035 (5)	−0.016 (4)	0.003 (4)	0.023 (4)
C19	0.040 (5)	0.062 (6)	0.015 (4)	0.005 (4)	0.004 (3)	0.002 (4)
C20	0.019 (3)	0.017 (3)	0.020 (3)	−0.003 (3)	0.009 (3)	0.000 (3)
C21	0.019 (3)	0.022 (3)	0.012 (3)	−0.002 (3)	0.002 (3)	0.003 (3)
C22	0.021 (3)	0.024 (3)	0.015 (3)	0.000 (3)	0.002 (3)	0.003 (3)
C23	0.021 (4)	0.033 (4)	0.014 (3)	−0.004 (3)	0.000 (3)	0.001 (3)
C24	0.025 (4)	0.029 (4)	0.023 (4)	−0.002 (3)	0.002 (3)	−0.006 (3)
C25	0.016 (3)	0.035 (4)	0.024 (4)	0.007 (3)	0.001 (3)	0.006 (3)
C26	0.031 (4)	0.030 (4)	0.019 (4)	−0.004 (3)	0.007 (3)	0.002 (3)
C27	0.027 (4)	0.027 (4)	0.029 (4)	0.003 (3)	0.014 (3)	0.009 (3)
C28	0.019 (3)	0.044 (4)	0.014 (3)	0.003 (3)	−0.003 (3)	0.009 (3)
C29	0.026 (4)	0.040 (4)	0.021 (4)	0.004 (3)	0.007 (3)	0.000 (3)
C111	0.023 (3)	0.021 (3)	0.013 (3)	−0.003 (3)	0.004 (3)	0.001 (3)
C112	0.061 (5)	0.019 (4)	0.028 (4)	−0.007 (3)	0.014 (4)	−0.006 (3)
C113	0.033 (4)	0.034 (4)	0.026 (4)	0.012 (3)	0.003 (3)	0.003 (3)
C211	0.027 (4)	0.021 (3)	0.018 (3)	0.005 (3)	0.002 (3)	0.000 (3)
C212	0.035 (4)	0.022 (4)	0.031 (4)	−0.005 (3)	0.001 (3)	0.001 (3)
C213	0.037 (4)	0.021 (4)	0.034 (4)	0.003 (3)	0.003 (4)	−0.001 (3)

### Geometric parameters (Å, º)

**Table:**

Pd1—N1	2.182 (5)	C15—C16	1.407 (11)
Pd1—Cl1	2.3585 (17)	C15—C19	1.414 (11)
Pd1—Se2	2.3898 (8)	C15—H15	0.9500
Pd1—Se1	2.4051 (9)	C16—C17	1.405 (11)
Pd2—N2	2.152 (5)	C16—H16	0.9500
Pd2—Cl2	2.3540 (17)	C17—C18	1.427 (11)
Pd2—Se2	2.3716 (8)	C17—H17	0.9500
Pd2—Se1	2.4166 (8)	C18—C19	1.426 (12)
Se1—C21	1.904 (6)	C18—H18	0.9500
Se2—C11	1.904 (6)	C19—H19	0.9500
N1—C112	1.475 (9)	C20—C24	1.431 (10)
N1—C113	1.483 (9)	C20—C21	1.442 (9)
N1—C111	1.500 (8)	C20—C211	1.497 (9)
N2—C212	1.470 (9)	C21—C22	1.417 (9)
N2—C211	1.488 (8)	C22—C23	1.431 (9)
N2—C213	1.499 (9)	C22—H22	0.9500
Fe1—C11	2.022 (6)	C23—C24	1.419 (10)
Fe1—C17	2.029 (7)	C23—H23	0.9500
Fe1—C18	2.031 (7)	C24—H24	0.9500
Fe1—C12	2.039 (7)	C25—C29	1.421 (10)
Fe1—C16	2.042 (7)	C25—C26	1.431 (10)
Fe1—C15	2.050 (7)	C25—H25	0.9500
Fe1—C13	2.049 (7)	C26—C27	1.432 (10)
Fe1—C10	2.055 (6)	C26—H26	0.9500
Fe1—C19	2.056 (7)	C27—C28	1.409 (10)
Fe1—C14	2.072 (7)	C27—H27	0.9500
Fe2—C20	2.042 (6)	C28—C29	1.422 (10)
Fe2—C21	2.043 (6)	C28—H28	0.9500
Fe2—C24	2.042 (7)	C29—H29	0.9500
Fe2—C28	2.043 (7)	C111—H11A	0.9900
Fe2—C25	2.046 (7)	C111—H11B	0.9900
Fe2—C23	2.046 (7)	C112—H11C	0.9800
Fe2—C27	2.052 (7)	C112—H11D	0.9800
Fe2—C26	2.053 (7)	C112—H11E	0.9800
Fe2—C29	2.054 (7)	C113—H11F	0.9800
Fe2—C22	2.056 (6)	C113—H11G	0.9800
C10—C11	1.436 (9)	C113—H11H	0.9800
C10—C14	1.443 (9)	C211—H21A	0.9900
C10—C111	1.494 (9)	C211—H21B	0.9900
C11—C12	1.415 (9)	C212—H21C	0.9800
C12—C13	1.404 (10)	C212—H21D	0.9800
C12—H12	0.9500	C212—H21E	0.9800
C13—C14	1.420 (10)	C213—H21F	0.9800
C13—H13	0.9500	C213—H21G	0.9800
C14—H14	0.9500	C213—H21H	0.9800
			
N1—Pd1—Cl1	92.99 (15)	C12—C13—C14	110.0 (6)
N1—Pd1—Se2	93.62 (14)	C12—C13—Fe1	69.5 (4)
Cl1—Pd1—Se2	173.39 (5)	C14—C13—Fe1	70.7 (4)
N1—Pd1—Se1	172.87 (15)	C12—C13—H13	125.0
Cl1—Pd1—Se1	93.59 (5)	C14—C13—H13	125.0
Se2—Pd1—Se1	79.80 (3)	Fe1—C13—H13	126.4
N2—Pd2—Cl2	92.05 (15)	C13—C14—C10	106.7 (6)
N2—Pd2—Se2	175.53 (15)	C13—C14—Fe1	69.0 (4)
Cl2—Pd2—Se2	91.22 (5)	C10—C14—Fe1	68.9 (4)
N2—Pd2—Se1	96.84 (14)	C13—C14—H14	126.7
Cl2—Pd2—Se1	171.10 (5)	C10—C14—H14	126.7
Se2—Pd2—Se1	79.92 (3)	Fe1—C14—H14	127.0
C21—Se1—Pd1	109.23 (19)	C16—C15—C19	108.7 (7)
C21—Se1—Pd2	95.12 (19)	C16—C15—Fe1	69.6 (4)
Pd1—Se1—Pd2	94.58 (3)	C19—C15—Fe1	70.1 (4)
C11—Se2—Pd2	105.90 (18)	C16—C15—H15	125.7
C11—Se2—Pd1	92.08 (19)	C19—C15—H15	125.7
Pd2—Se2—Pd1	96.17 (3)	Fe1—C15—H15	126.2
C112—N1—C113	109.7 (6)	C17—C16—C15	108.6 (7)
C112—N1—C111	108.0 (5)	C17—C16—Fe1	69.3 (4)
C113—N1—C111	108.3 (5)	C15—C16—Fe1	70.2 (4)
C112—N1—Pd1	106.7 (4)	C17—C16—H16	125.7
C113—N1—Pd1	109.3 (4)	C15—C16—H16	125.7
C111—N1—Pd1	114.8 (4)	Fe1—C16—H16	126.4
C212—N2—C211	109.4 (5)	C16—C17—C18	107.5 (7)
C212—N2—C213	108.4 (5)	C16—C17—Fe1	70.3 (4)
C211—N2—C213	106.8 (5)	C18—C17—Fe1	69.5 (4)
C212—N2—Pd2	107.3 (4)	C16—C17—H17	126.2
C211—N2—Pd2	114.3 (4)	C18—C17—H17	126.2
C213—N2—Pd2	110.4 (4)	Fe1—C17—H17	125.5
C11—Fe1—C17	122.5 (3)	C17—C18—C19	108.1 (7)
C11—Fe1—C18	160.0 (3)	C17—C18—Fe1	69.3 (4)
C17—Fe1—C18	41.1 (3)	C19—C18—Fe1	70.5 (4)
C11—Fe1—C12	40.8 (2)	C17—C18—H18	126.0
C17—Fe1—C12	104.1 (3)	C19—C18—H18	126.0
C18—Fe1—C12	123.1 (3)	Fe1—C18—H18	125.7
C11—Fe1—C16	106.5 (3)	C15—C19—C18	107.1 (7)
C17—Fe1—C16	40.4 (3)	C15—C19—Fe1	69.6 (4)
C18—Fe1—C16	68.2 (3)	C18—C19—Fe1	68.6 (4)
C12—Fe1—C16	118.0 (3)	C15—C19—H19	126.4
C11—Fe1—C15	121.2 (3)	C18—C19—H19	126.4
C17—Fe1—C15	68.1 (3)	Fe1—C19—H19	126.9
C18—Fe1—C15	68.1 (3)	C24—C20—C21	105.9 (6)
C12—Fe1—C15	154.1 (3)	C24—C20—C211	129.6 (6)
C16—Fe1—C15	40.2 (3)	C21—C20—C211	124.4 (6)
C11—Fe1—C13	67.9 (3)	C24—C20—Fe2	69.5 (4)
C17—Fe1—C13	118.4 (3)	C21—C20—Fe2	69.3 (3)
C18—Fe1—C13	107.4 (3)	C211—C20—Fe2	127.9 (4)
C12—Fe1—C13	40.2 (3)	C22—C21—C20	109.4 (6)
C16—Fe1—C13	152.7 (3)	C22—C21—Se1	132.1 (5)
C15—Fe1—C13	165.3 (3)	C20—C21—Se1	118.5 (5)
C11—Fe1—C10	41.2 (2)	C22—C21—Fe2	70.3 (4)
C17—Fe1—C10	161.4 (3)	C20—C21—Fe2	69.3 (3)
C18—Fe1—C10	157.0 (3)	Se1—C21—Fe2	126.7 (3)
C12—Fe1—C10	68.9 (3)	C21—C22—C23	107.4 (6)
C16—Fe1—C10	126.4 (3)	C21—C22—Fe2	69.3 (4)
C15—Fe1—C10	110.3 (3)	C23—C22—Fe2	69.2 (4)
C13—Fe1—C10	68.1 (3)	C21—C22—H22	126.3
C11—Fe1—C19	157.0 (3)	C23—C22—H22	126.3
C17—Fe1—C19	68.8 (3)	Fe2—C22—H22	126.8
C18—Fe1—C19	40.8 (3)	C24—C23—C22	108.0 (6)
C12—Fe1—C19	161.9 (3)	C24—C23—Fe2	69.5 (4)
C16—Fe1—C19	68.0 (3)	C22—C23—Fe2	70.0 (4)
C15—Fe1—C19	40.3 (3)	C24—C23—H23	126.0
C13—Fe1—C19	127.3 (3)	C22—C23—H23	126.0
C10—Fe1—C19	122.8 (3)	Fe2—C23—H23	126.1
C11—Fe1—C14	68.9 (2)	C23—C24—C20	109.2 (6)
C17—Fe1—C14	154.2 (3)	C23—C24—Fe2	69.8 (4)
C18—Fe1—C14	121.0 (3)	C20—C24—Fe2	69.5 (4)
C12—Fe1—C14	68.5 (3)	C23—C24—H24	125.4
C16—Fe1—C14	165.0 (3)	C20—C24—H24	125.4
C15—Fe1—C14	128.9 (3)	Fe2—C24—H24	126.9
C13—Fe1—C14	40.3 (3)	C29—C25—C26	108.9 (6)
C10—Fe1—C14	40.9 (3)	C29—C25—Fe2	70.0 (4)
C19—Fe1—C14	110.3 (3)	C26—C25—Fe2	69.8 (4)
C20—Fe2—C21	41.3 (2)	C29—C25—H25	125.5
C20—Fe2—C24	41.0 (3)	C26—C25—H25	125.5
C21—Fe2—C24	68.3 (3)	Fe2—C25—H25	126.2
C20—Fe2—C28	157.6 (3)	C27—C26—C25	106.7 (6)
C21—Fe2—C28	159.1 (3)	C27—C26—Fe2	69.6 (4)
C24—Fe2—C28	121.4 (3)	C25—C26—Fe2	69.3 (4)
C20—Fe2—C25	107.8 (3)	C27—C26—H26	126.7
C21—Fe2—C25	124.4 (3)	C25—C26—H26	126.7
C24—Fe2—C25	123.3 (3)	Fe2—C26—H26	126.1
C28—Fe2—C25	68.0 (3)	C28—C27—C26	108.4 (6)
C20—Fe2—C23	69.3 (3)	C28—C27—Fe2	69.5 (4)
C21—Fe2—C23	68.3 (3)	C26—C27—Fe2	69.6 (4)
C24—Fe2—C23	40.6 (3)	C28—C27—H27	125.8
C28—Fe2—C23	106.1 (3)	C26—C27—H27	125.8
C25—Fe2—C23	158.5 (3)	Fe2—C27—H27	126.6
C20—Fe2—C27	160.7 (3)	C27—C28—C29	108.9 (6)
C21—Fe2—C27	124.2 (3)	C27—C28—Fe2	70.2 (4)
C24—Fe2—C27	157.1 (3)	C29—C28—Fe2	70.1 (4)
C28—Fe2—C27	40.3 (3)	C27—C28—H28	125.5
C25—Fe2—C27	68.2 (3)	C29—C28—H28	125.5
C23—Fe2—C27	121.6 (3)	Fe2—C28—H28	125.7
C20—Fe2—C26	123.8 (3)	C25—C29—C28	107.1 (6)
C21—Fe2—C26	108.8 (3)	C25—C29—Fe2	69.4 (4)
C24—Fe2—C26	160.2 (3)	C28—C29—Fe2	69.3 (4)
C28—Fe2—C26	68.4 (3)	C25—C29—H29	126.4
C25—Fe2—C26	40.9 (3)	C28—C29—H29	126.4
C23—Fe2—C26	158.3 (3)	Fe2—C29—H29	126.4
C27—Fe2—C26	40.8 (3)	C10—C111—N1	111.8 (5)
C20—Fe2—C29	121.8 (3)	C10—C111—H11A	109.3
C21—Fe2—C29	159.5 (3)	N1—C111—H11A	109.3
C24—Fe2—C29	106.6 (3)	C10—C111—H11B	109.3
C28—Fe2—C29	40.6 (3)	N1—C111—H11B	109.3
C25—Fe2—C29	40.6 (3)	H11A—C111—H11B	107.9
C23—Fe2—C29	121.6 (3)	N1—C112—H11C	109.5
C27—Fe2—C29	68.2 (3)	N1—C112—H11D	109.5
C26—Fe2—C29	68.8 (3)	H11C—C112—H11D	109.5
C20—Fe2—C22	69.4 (3)	N1—C112—H11E	109.5
C21—Fe2—C22	40.5 (2)	H11C—C112—H11E	109.5
C24—Fe2—C22	68.5 (3)	H11D—C112—H11E	109.5
C28—Fe2—C22	122.3 (3)	N1—C113—H11F	109.5
C25—Fe2—C22	159.8 (3)	N1—C113—H11G	109.5
C23—Fe2—C22	40.8 (3)	H11F—C113—H11G	109.5
C27—Fe2—C22	107.5 (3)	N1—C113—H11H	109.5
C26—Fe2—C22	123.0 (3)	H11F—C113—H11H	109.5
C29—Fe2—C22	158.1 (3)	H11G—C113—H11H	109.5
C11—C10—C14	107.1 (6)	N2—C211—C20	111.3 (5)
C11—C10—C111	121.9 (6)	N2—C211—H21A	109.4
C14—C10—C111	130.9 (6)	C20—C211—H21A	109.4
C11—C10—Fe1	68.2 (4)	N2—C211—H21B	109.4
C14—C10—Fe1	70.2 (4)	C20—C211—H21B	109.4
C111—C10—Fe1	129.7 (5)	H21A—C211—H21B	108.0
C12—C11—C10	108.7 (6)	N2—C212—H21C	109.5
C12—C11—Se2	133.6 (5)	N2—C212—H21D	109.5
C10—C11—Se2	117.8 (4)	H21C—C212—H21D	109.5
C12—C11—Fe1	70.2 (4)	N2—C212—H21E	109.5
C10—C11—Fe1	70.6 (4)	H21C—C212—H21E	109.5
Se2—C11—Fe1	124.4 (3)	H21D—C212—H21E	109.5
C13—C12—C11	107.5 (6)	N2—C213—H21F	109.5
C13—C12—Fe1	70.3 (4)	N2—C213—H21G	109.5
C11—C12—Fe1	69.0 (4)	H21F—C213—H21G	109.5
C13—C12—H12	126.3	N2—C213—H21H	109.5
C11—C12—H12	126.3	H21F—C213—H21H	109.5
Fe1—C12—H12	126.0	H21G—C213—H21H	109.5
			
C14—C10—C11—C12	−0.6 (7)	C24—C20—C21—Fe2	60.2 (4)
C111—C10—C11—C12	175.7 (6)	C211—C20—C21—Fe2	−122.5 (6)
Fe1—C10—C11—C12	−60.2 (4)	C20—C21—C22—C23	−0.5 (7)
C14—C10—C11—Se2	179.1 (4)	Se1—C21—C22—C23	179.0 (5)
C111—C10—C11—Se2	−4.6 (8)	Fe2—C21—C22—C23	−58.9 (4)
Fe1—C10—C11—Se2	119.5 (4)	C20—C21—C22—Fe2	58.4 (4)
C14—C10—C11—Fe1	59.6 (4)	Se1—C21—C22—Fe2	−122.1 (5)
C111—C10—C11—Fe1	−124.1 (6)	C21—C22—C23—C24	−0.4 (7)
C10—C11—C12—C13	0.4 (7)	Fe2—C22—C23—C24	−59.3 (4)
Se2—C11—C12—C13	−179.2 (5)	C21—C22—C23—Fe2	59.0 (4)
Fe1—C11—C12—C13	−60.0 (5)	C22—C23—C24—C20	1.2 (7)
C10—C11—C12—Fe1	60.4 (4)	Fe2—C23—C24—C20	−58.4 (5)
Se2—C11—C12—Fe1	−119.2 (6)	C22—C23—C24—Fe2	59.6 (4)
C11—C12—C13—C14	−0.1 (8)	C21—C20—C24—C23	−1.5 (7)
Fe1—C12—C13—C14	−59.3 (5)	C211—C20—C24—C23	−178.5 (6)
C11—C12—C13—Fe1	59.2 (4)	Fe2—C20—C24—C23	58.6 (5)
C12—C13—C14—C10	−0.3 (8)	C21—C20—C24—Fe2	−60.1 (4)
Fe1—C13—C14—C10	−58.8 (4)	C211—C20—C24—Fe2	122.8 (7)
C12—C13—C14—Fe1	58.5 (5)	C29—C25—C26—C27	0.5 (8)
C11—C10—C14—C13	0.5 (7)	Fe2—C25—C26—C27	59.8 (5)
C111—C10—C14—C13	−175.3 (6)	C29—C25—C26—Fe2	−59.3 (5)
Fe1—C10—C14—C13	58.9 (5)	C25—C26—C27—C28	−0.7 (7)
C11—C10—C14—Fe1	−58.3 (4)	Fe2—C26—C27—C28	58.9 (5)
C111—C10—C14—Fe1	125.8 (7)	C25—C26—C27—Fe2	−59.6 (5)
C19—C15—C16—C17	−0.5 (8)	C26—C27—C28—C29	0.7 (8)
Fe1—C15—C16—C17	58.8 (5)	Fe2—C27—C28—C29	59.6 (5)
C19—C15—C16—Fe1	−59.4 (5)	C26—C27—C28—Fe2	−59.0 (5)
C15—C16—C17—C18	0.4 (8)	C26—C25—C29—C28	−0.1 (8)
Fe1—C16—C17—C18	59.8 (5)	Fe2—C25—C29—C28	−59.3 (5)
C15—C16—C17—Fe1	−59.4 (5)	C26—C25—C29—Fe2	59.2 (5)
C16—C17—C18—C19	−0.1 (8)	C27—C28—C29—C25	−0.3 (8)
Fe1—C17—C18—C19	60.2 (5)	Fe2—C28—C29—C25	59.4 (5)
C16—C17—C18—Fe1	−60.3 (5)	C27—C28—C29—Fe2	−59.7 (5)
C16—C15—C19—C18	0.5 (8)	C11—C10—C111—N1	−69.5 (8)
Fe1—C15—C19—C18	−58.6 (5)	C14—C10—C111—N1	105.8 (8)
C16—C15—C19—Fe1	59.1 (5)	Fe1—C10—C111—N1	−156.5 (5)
C17—C18—C19—C15	−0.2 (8)	C112—N1—C111—C10	174.3 (6)
Fe1—C18—C19—C15	59.2 (5)	C113—N1—C111—C10	−67.0 (7)
C17—C18—C19—Fe1	−59.4 (5)	Pd1—N1—C111—C10	55.5 (6)
C24—C20—C21—C22	1.2 (7)	C212—N2—C211—C20	−59.8 (7)
C211—C20—C21—C22	178.5 (6)	C213—N2—C211—C20	−177.0 (5)
Fe2—C20—C21—C22	−59.0 (4)	Pd2—N2—C211—C20	60.7 (6)
C24—C20—C21—Se1	−178.4 (4)	C24—C20—C211—N2	105.3 (7)
C211—C20—C21—Se1	−1.1 (8)	C21—C20—C211—N2	−71.2 (8)
Fe2—C20—C21—Se1	121.4 (4)	Fe2—C20—C211—N2	−160.8 (4)

### Hydrogen-bond geometry (Å, º)

**Table:**

*D*—H···*A*	*D*—H	H···*A*	*D*···*A*	*D*—H···*A*
C113—H11*F*···Cl1	0.98	2.70	3.347 (8)	124
C212—H21*D*···Cl2	0.98	2.75	3.394 (8)	124
C213—H21*H*···Cl2	0.98	2.74	3.384 (8)	124
C22—H22···Cl1	0.95	2.82	3.537 (7)	133
