# Supplementary figures and images for: Crystal structure of bis­{μ-2-[(di­methyl­amino)­meth­yl]ferrocene­seleno­lato}bis[chlorido­palladium(II)]
Source: Acta Crystallogr Sect E Struct Rep Online. 2014 Sep 3;70(Pt 10):m343–4. doi: 10.1107/S1600536814019503 (PMC4257227; doi:10.1107/S1600536814019503)

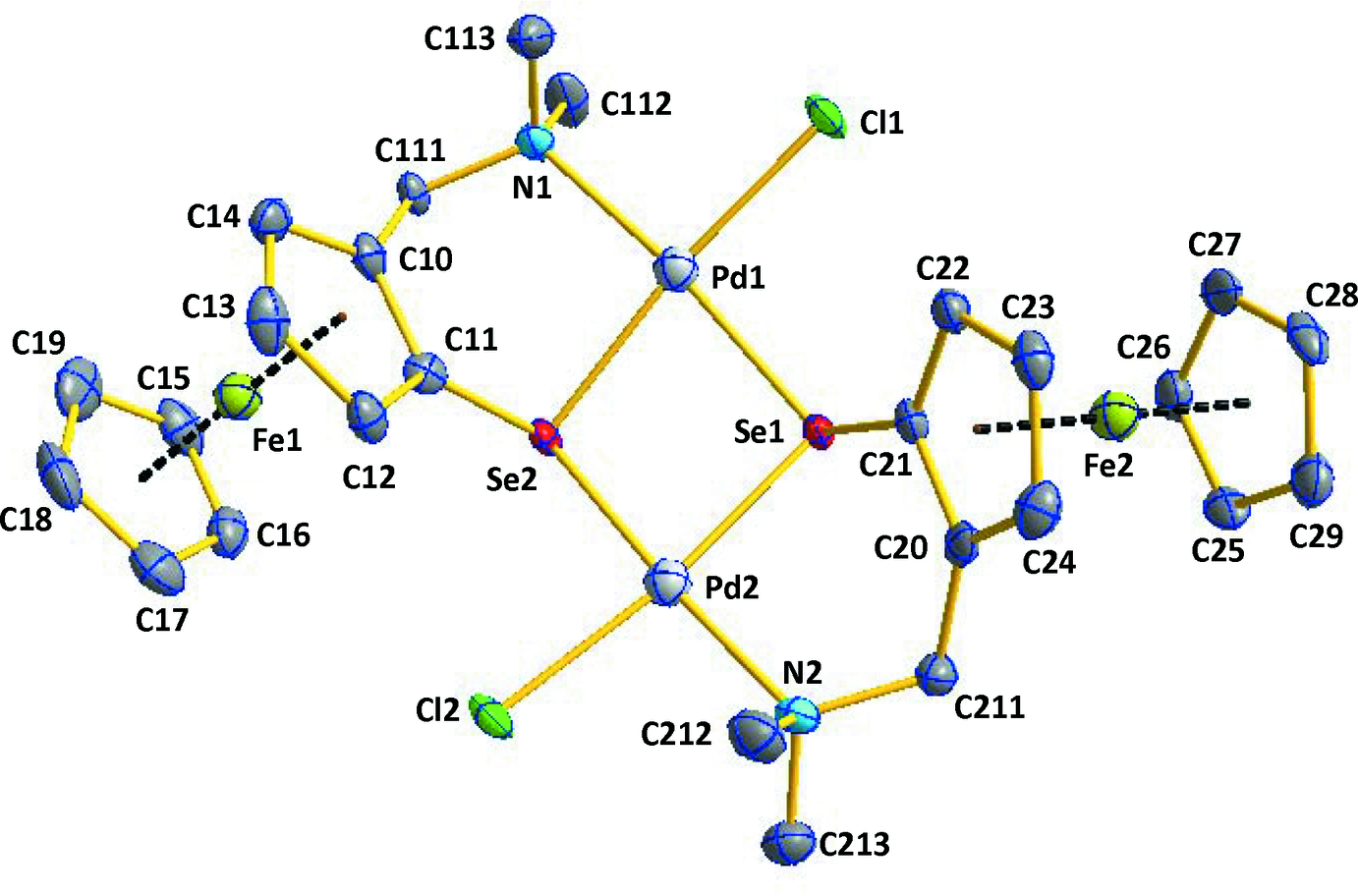

Supplement: Supplementary file 3 [file e-70-0m343-fig1.tif]
